# Supplementary material for: Mechanosensing regulates tissue repair program in macrophages
Source: Sci Adv. 2024 Mar 13;10(11):eadk6906. doi: 10.1126/sciadv.adk6906 (PMC10936955; doi:10.1126/sciadv.adk6906)
Supplement: Supplementary file 1 — Figs. S1 to S5 Table S1 Legend for movies S1 to S4 [file sciadv.adk6906_sm.pdf]

Science Advances
AAAS

Supplementary Materials for

Mechanosensing regulates tissue repair program in macrophages

Matthew L. Meizlish *et al.*

Corresponding author: Ruslan Medzhitov, ruslan.medzhitov@yale.edu

Sci. Adv. **10**, eadk6906 (2024)
DOI: 10.1126/sciadv.adk6906

The PDF file includes:

Figs. S1 to S5
Table S1
Legend for movies S1 to S4

Other Supplementary Material for this manuscript includes the following:

Movies S1 to S4

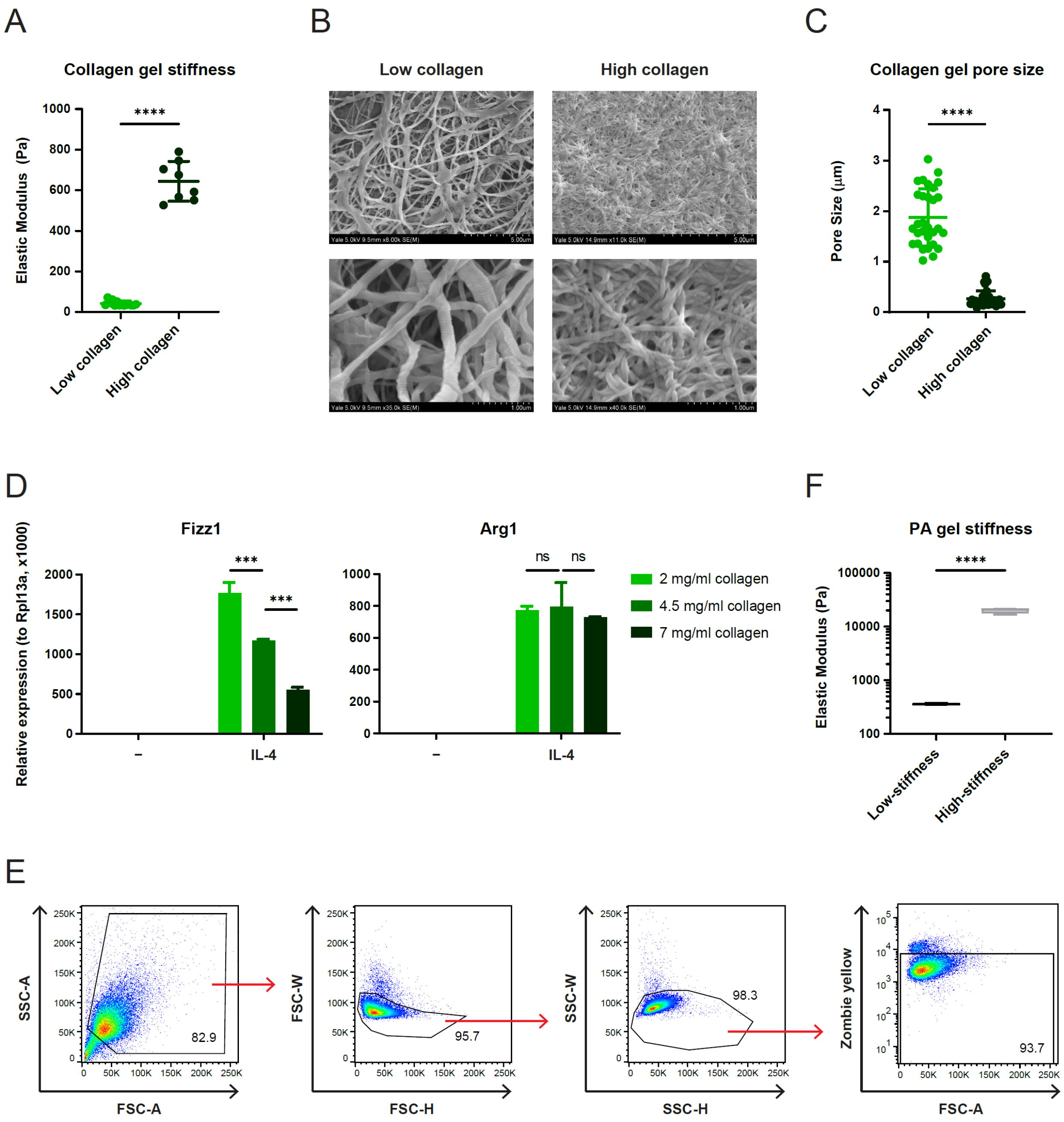

Fig. S1. Mechanical properties of collagen gels and PA hydrogels

(A) Stiffness of low- and high-collagen gels, measured by rheology. **(B)** Scanning electron microscopy (SEM) images of low- and high-collagen gels at lower (above) and higher (below) magnification. **(C)** Pore sizes of low- and high-collagen gels quantified in SEM images. **(D)** Relative gene expression in BMDMs cultured in indicated concentration of collagen gels with or without IL-4. **(E)** Gating strategy for flow cytometry analysis of FIZZ1 and ARG1 protein expression in BMDM. **(F)** Stiffness of low- and high-stiffness PA gels, measured by rheology. Data are represented as mean ± SD. Student's t test (A, B, and F) and two-way ANOVA (D) are used for statistical analysis. *** $p < 0.001$, **** $p < 0.0001$. ns; not significant.

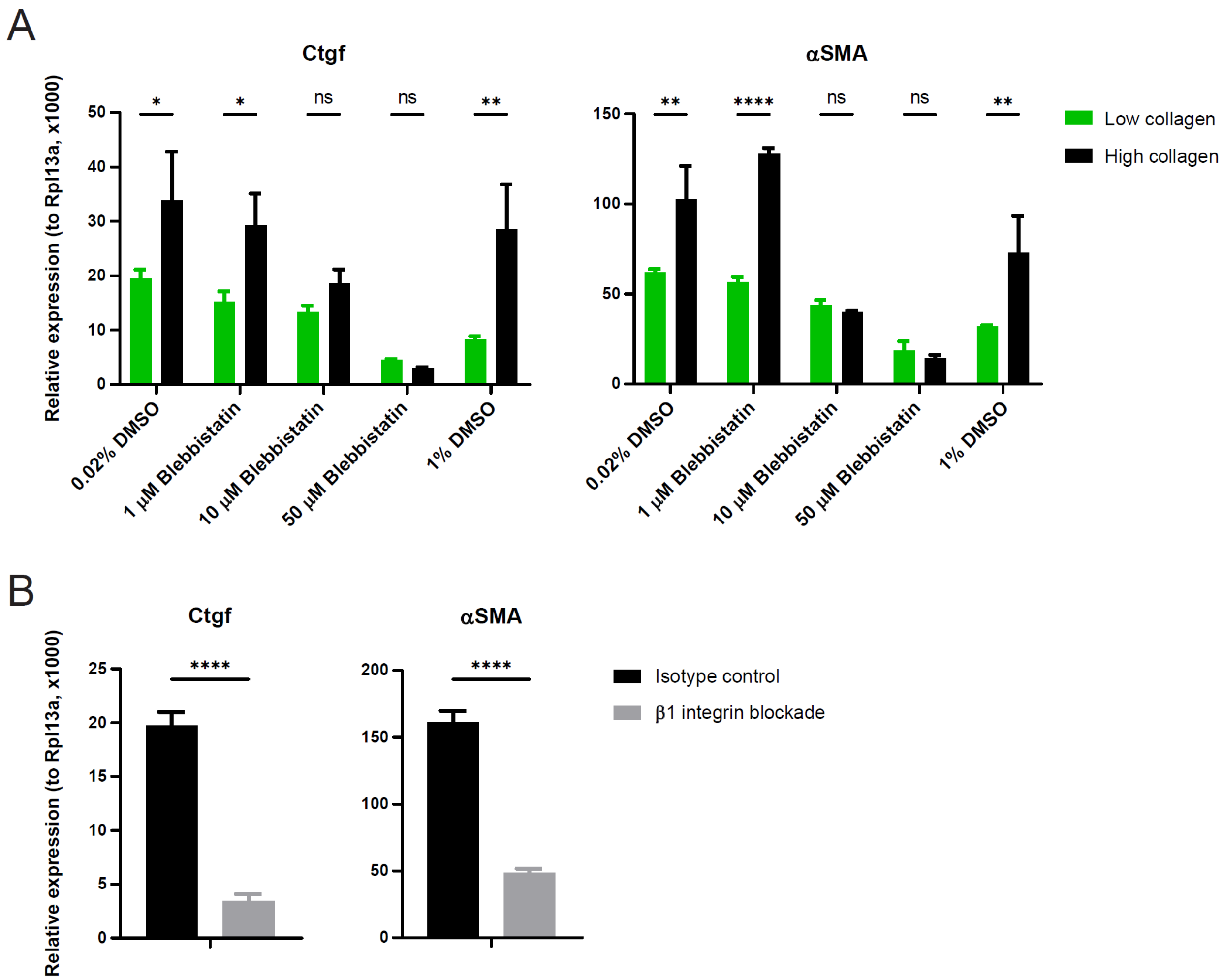

Fig. S2. Fibroblast mechanosensing is integrin- and non-muscle myosin II-dependent.

(A) Relative gene expression in MEFs cultured in low- or high-collagen gels with the indicated doses of blebbistatin or DMSO (equivalent to the amount of DMSO in the lowest and highest concentrations of blebbistatin). **(B)** Relative gene expression in MEFs cultured in 3D low-collagen gels with β1 integrin-blocking antibody or isotype control antibody. Data are represented as mean ± SD. Two-way ANOVA (A) and Student's t test (B) were used for statistical analysis. *$p < 0.05$, ** $p < 0.01$, **** $p < 0.0001$. ns; not significant.

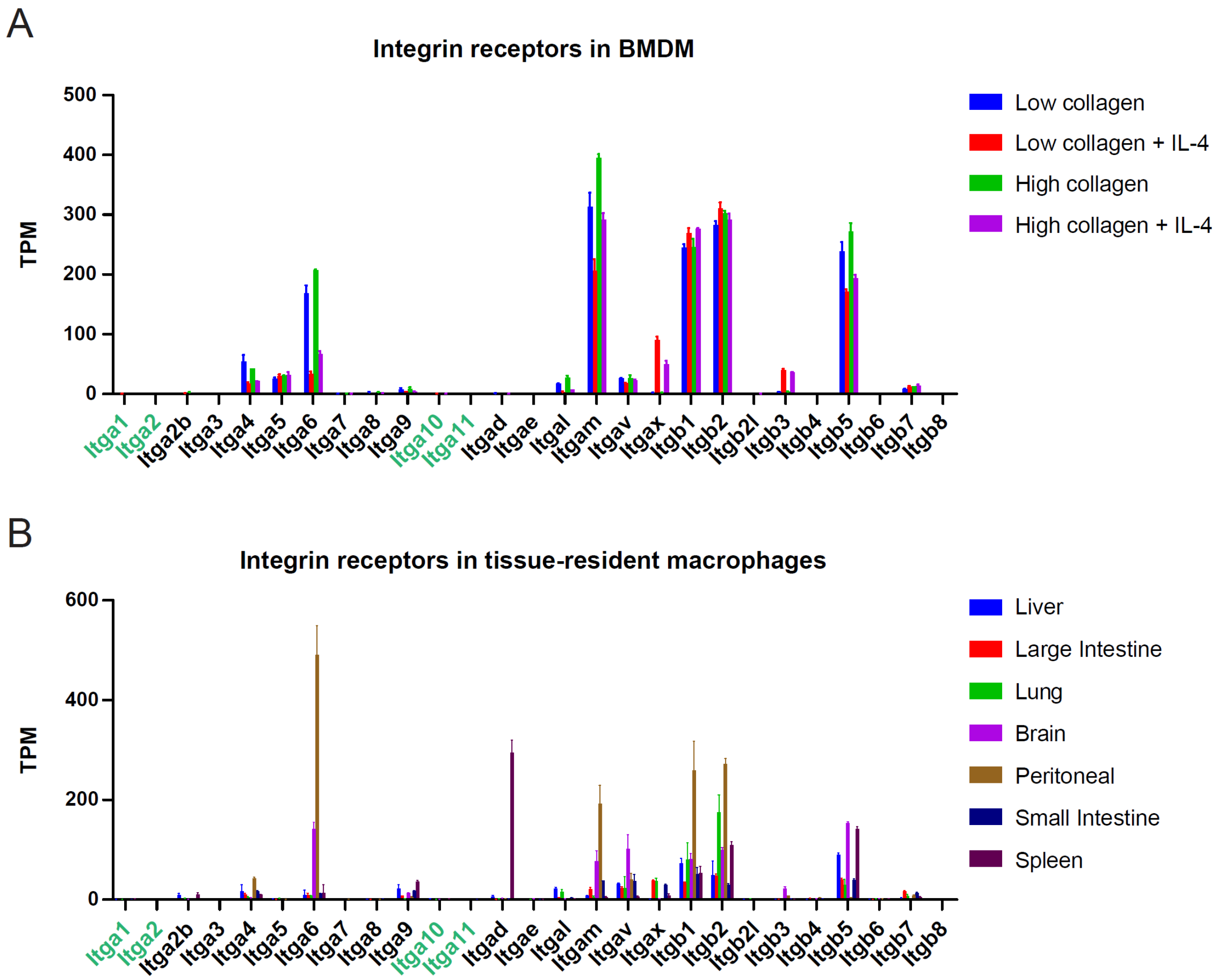

Fig. S3. Macrophages show minimal expression of collagen-binding integrins.

(A) Normalized gene expression levels (transcripts per million, TPM) of all integrin alpha and beta chains in BMDMs, from RNA-seq analysis of BMDMs cultured in 3D low- or high-collagen gels, with or without IL-4 stimulation. **(B)** Normalized gene expression levels (TPM) of all integrin alpha and beta chains in various tissue-resident macrophage populations, based on reanalysis of RNA-seq data from Lavin et al.[56] Alpha chains of collagen-binding integrins are highlighted in green (Itga1, Itga2, Itga10, Itga11). Data are represented as mean ± SD.

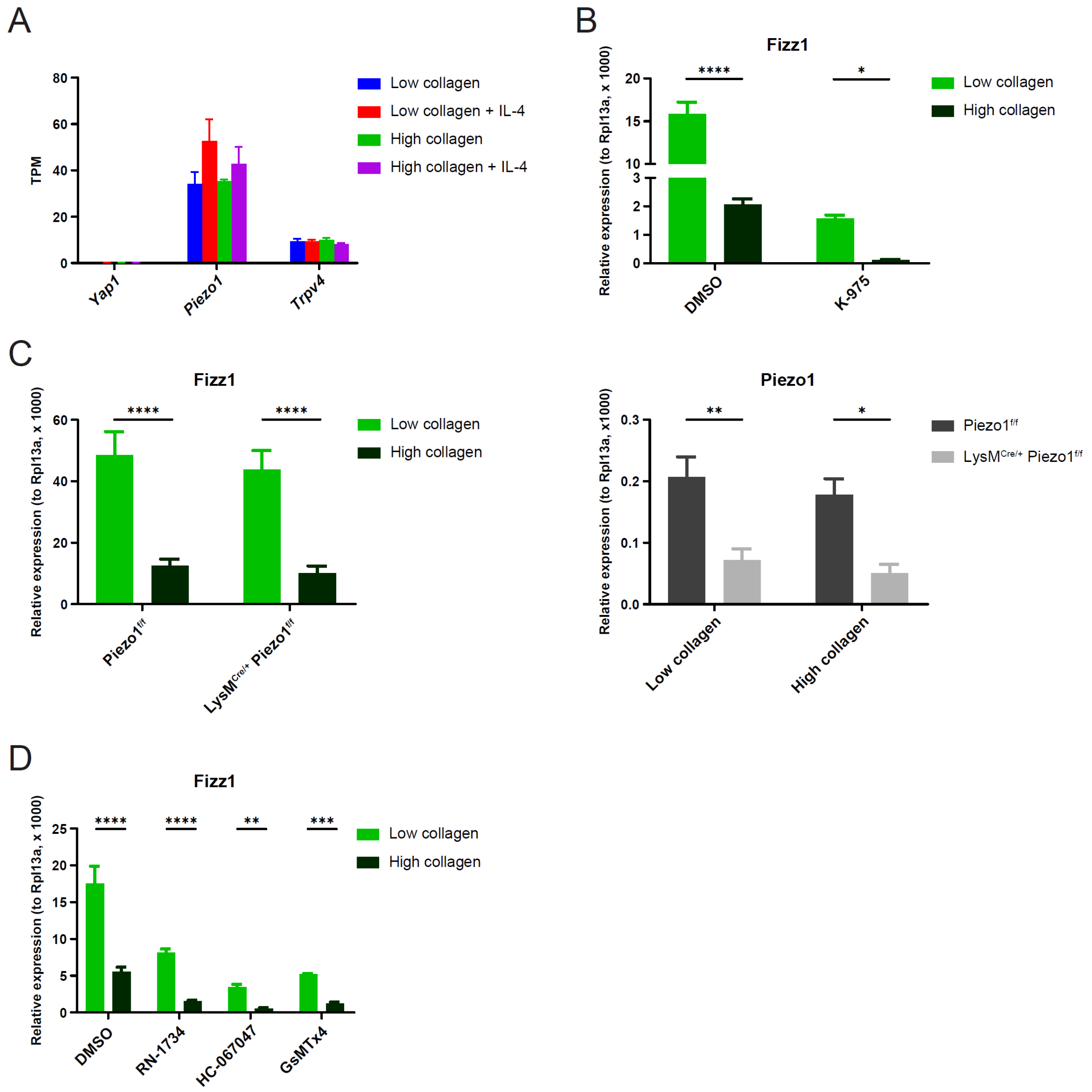

Fig. S4. YAP signaling and mechanosensitive ion channels are not required for macrophage mechanosensing.

(A) TPM of *Yap1*, *Piezo1* and *Trpv4* from RNA-seq analysis of BMDMs cultured in 3D low- or high- collagen gels with or without IL-4 stimulation. **(B)** Relative gene expression in BMDMs treated with control or K-975 (TEAD inhibitor) and cultured in low- or high-collagen gels in the presence of IL-4. **(C)** Relative gene expression in BMDMs derived from Piezo1$^{f/f}$ or LysM$^{Cre/+}$ Piezo1$^{f/f}$ mice, cultured in low- or high-collagen gels in the presence of IL-4. **(D)** Relative gene expression in BMDMs treated with control, RN-1734 or HC-067047 (TRPV4 inhibitors), or GsMTx4 (mechanosensitive ion channel inhibitor) and cultured in low- or high-collagen gels in the presence of IL-4. Data are represented as mean ± SD. Two-way ANOVA was used for statistical analysis. *$p < 0.05$, ** $p < 0.01$, *** $p < 0.001$, **** $p < 0.0001$.

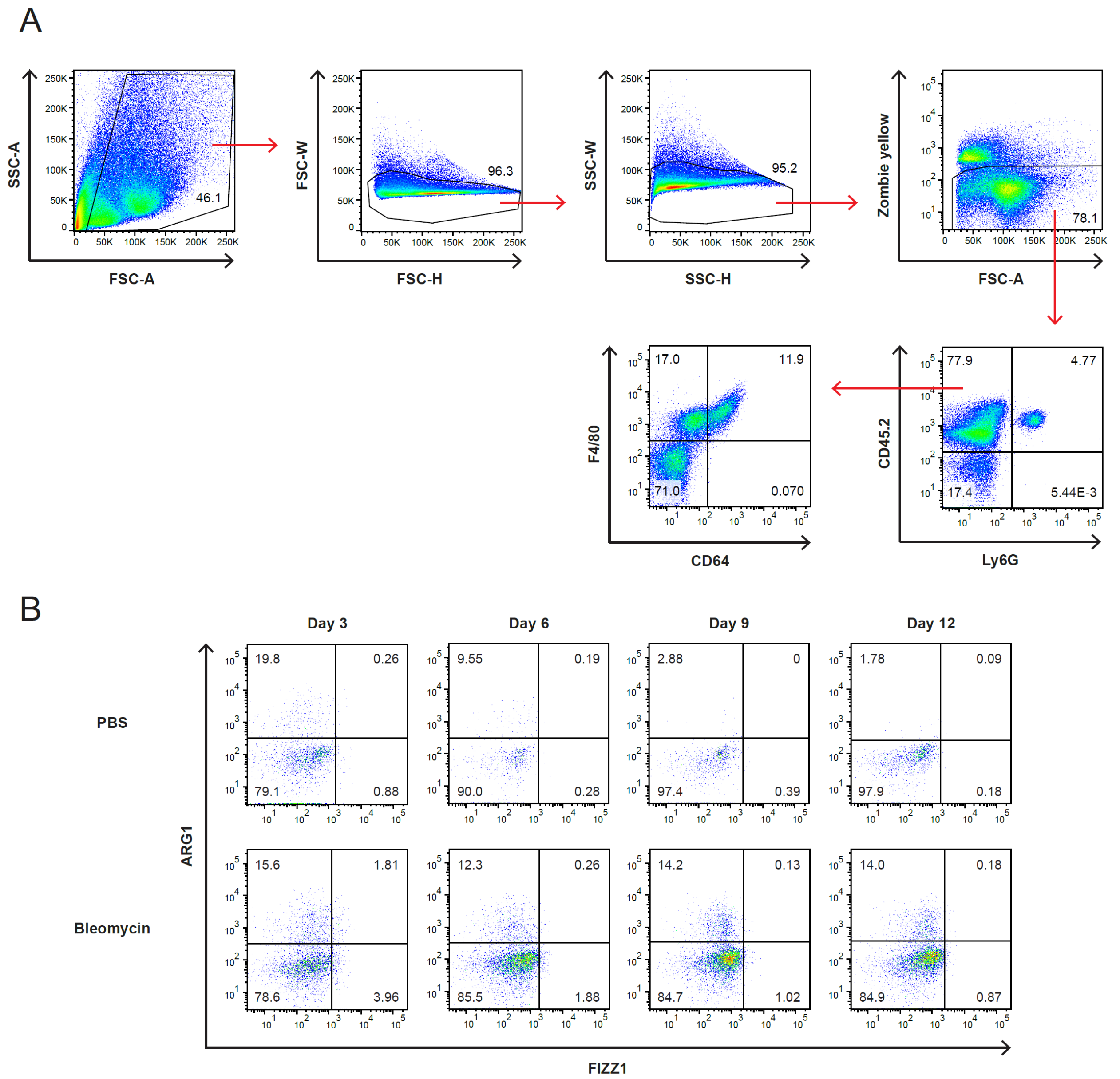

Fig. S5. Mechanosensitive gene expression program is induced during tissue repair *in vivo*. **(A)** Gating strategy for flow cytometry analysis of FIZZ1 and ARG1 protein expression in mouse lung macrophages. **(B)** FIZZ1 and ARG1 protein expression in lung macrophages in PBS- or bleomycin-treated mice, analyzed by flow cytometry. Representative plots of $CD64^+$ $F4/80^+$ cells following the gating strategy in (A) are shown.

Gene name		Sequence (5' to 3')
Arg1	Forward	CTGGTGTGGTGGCAGAGG
	Reverse	TGGCCAGAGATGCTTCCAAC
Mrc1	Forward	AAAGGGACGTTTCGGTGGAC
	Reverse	CACTCCGGTTTTCATGGCAAC
Fizz1	Forward	GATGAAGACTACAACTTGTTCC
	Reverse	AGGGATAGTTAGCTGGATTG
Rnase2a	Forward	TCCACGGGAGCCACAAAG
	Reverse	GAGGCAAGCATTAGGACATGTC
Ear2	Forward	TCCACGGGAGCCACAAAG
	Reverse	GAGGCAAGCATTAGGACAAGTC
Ym1	Forward	CCCTACAATTAGTACTGGCCCAC
	Reverse	CCTCAGTGGCTCCTTCATTCAG
Fn1	Forward	CAACCTCTGCAGACCTACCC
	Reverse	ACTGGATGGGGTGGGAATTG
Ccl24	Forward	AGCATCTGTCCCAAGGCAG
	Reverse	TGTATGTGCCTCTGAACCCAC
Ctgf	Forward	AGGGCCTCTTCTGCGATTTC
	Reverse	GACCCACCGAAGACACAGG
Acta2	Forward	ATCACCATTGGAAACGAACGC
	Reverse	TAGGTGGTTTCGTGGATGCC
Col1a1	Forward	ACGAGATGGCATCCCTGGA
	Reverse	GCCATAGGACATCTGGGAAGC
Talin1	Forward	TCTACCATGGTGTACGACGC
	Reverse	GACAGAAAGAGCCCAAAGTCG
Rpl13a	Forward	GAAGGAAAAGGCCAAGATGCAC
	Reverse	TGAGGACCTCTGTGAACTTGC

Table S1. List of primers used for qPCR analysis

Movie S1. Macrophages in 3D low-collagen gels

Movie S2. Macrophages in 3D high-collagen gels

Movie S3. Macrophages in 3D low-collagen gels without CSF1

Movie S4. Macrophages in 3D low-collagen gels with CSF1
